# Physiological, Biochemical, and Molecular Mechanisms of Heat Stress Tolerance in Plants

**DOI:** 10.3390/ijms14059643

**Published:** 2013-05-03

**Authors:** Mirza Hasanuzzaman, Kamrun Nahar, Md. Mahabub Alam, Rajib Roychowdhury, Masayuki Fujita

**Affiliations:** 1 Department of Agronomy, Faculty of Agriculture, Sher-e-Bangla Agricultural University, Dhaka 1207, Bangladesh; 2 Laboratory of Plant Stress Responses, Department of Applied Biological Science, Faculty of Agriculture, Kagawa University, Miki-cho, Kita-gun, Kagawa 761-0795, Japan; E-Mails: knahar84@yahoo.com (K.N.); shamim1983@yahoo.com (M.M.A.); 3 Department of Agricultural Botany, Faculty of Agriculture, Sher-e-Bangla Agricultural University, Sher-e-Bangla Nagar, Dhaka 1207, Bangladesh; 4 Department of Biotechnology, Visva-Bharati University, Santiniketan 731235, West Bengal, India; E-Mail: mhzsauag@yahoo.com

**Keywords:** abiotic stress, antioxidant defense, climate change, high temperature, heat shock proteins, oxidative stress, plant omics, stress signaling

## Abstract

High temperature (HT) stress is a major environmental stress that limits plant growth, metabolism, and productivity worldwide. Plant growth and development involve numerous biochemical reactions that are sensitive to temperature. Plant responses to HT vary with the degree and duration of HT and the plant type. HT is now a major concern for crop production and approaches for sustaining high yields of crop plants under HT stress are important agricultural goals. Plants possess a number of adaptive, avoidance, or acclimation mechanisms to cope with HT situations. In addition, major tolerance mechanisms that employ ion transporters, proteins, osmoprotectants, antioxidants, and other factors involved in signaling cascades and transcriptional control are activated to offset stress-induced biochemical and physiological alterations. Plant survival under HT stress depends on the ability to perceive the HT stimulus, generate and transmit the signal, and initiate appropriate physiological and biochemical changes. HT-induced gene expression and metabolite synthesis also substantially improve tolerance. The physiological and biochemical responses to heat stress are active research areas, and the molecular approaches are being adopted for developing HT tolerance in plants. This article reviews the recent findings on responses, adaptation, and tolerance to HT at the cellular, organellar, and whole plant levels and describes various approaches being taken to enhance thermotolerance in plants.

## 1. Introduction

Among the ever-changing components of the environment, the constantly rising ambient temperature is considered one of the most detrimental stresses. The global air temperature is predicted to rise by 0.2 °C per decade, which will lead to temperatures 1.8–4.0 °C higher than the current level by 2100 [1]. This prediction is creating apprehension among scientists, as heat stress has known effects on the life processes of organisms, acting directly or through the modification of surrounding environmental components. Plants, in particular, as sessile organisms, cannot move to more favorable environments; consequently, plant growth and developmental processes are substantially affected, often lethally, by high temperature (HT) stress [2,3].

**Figure 1:**
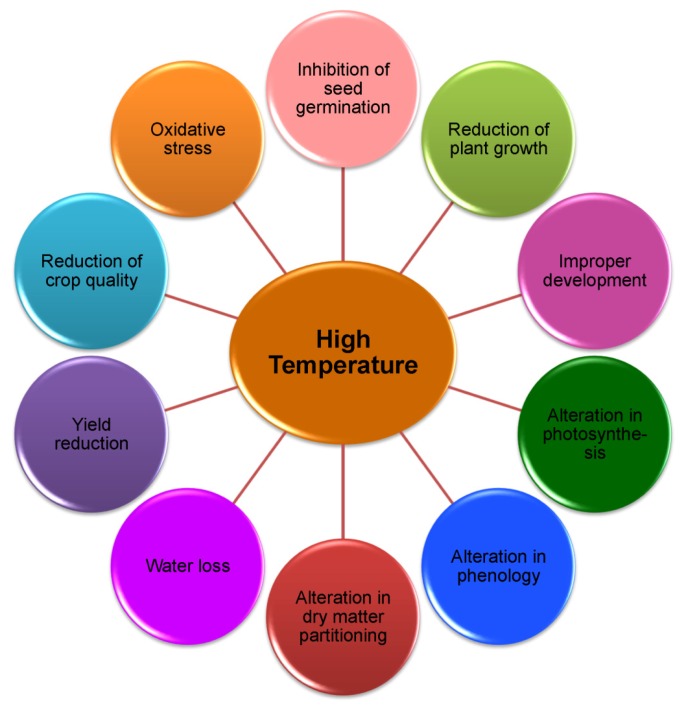
Major effects of high temperature on plants.

Heat stress causes multifarious, and often adverse, alterations in plant growth, development, physiological processes, and yield [4,5] (Figure 1). One of the major consequences of HT stress is the excess generation of reactive oxygen species (ROS), which leads to oxidative stress [4,5]. Plants continuously struggle for survival under various environmental stress conditions including HT. A plant is able, to some extent, to tolerate heat stress by physical changes within the plant body and frequently by creating signals for changing metabolism. Plants alter their metabolism in various ways in response to HT, particularly by producing compatible solutes that are able to organize proteins and cellular structures, maintain cell turgor by osmotic adjustment, and modify the antioxidant system to re-establish the cellular redox balance and homeostasis [6–8]. At the molecular level, heat stress causes alterations in expression of genes involved in direct protection from HT stress [9,10]. These include genes responsible for the expression of osmoprotectants, detoxifying enzymes, transporters, and regulatory proteins [11,12]. In conditions such as HT, modification of physiological and biochemical processes by gene expression changes gradually leads to the development of heat tolerance in the form of acclimation, or in the ideal case, to adaptation [13,14]. In recent times, exogenous applications of protectants in the form of osmoprotectants (proline, Pro; glycine betaine, GB; trehalose, Tre, *etc.*), phytohormones (abscisic acid, ABA; gibberellic acids, GA; jasmonic acids, JA; brassinosterioids, BR; salicylic acid, SA; *etc.*), signaling molecules (e.g., nitric oxide, NO), polyamines (putrescine, Put; spermidine, Spd and spermine, Spm), trace elements (selenium, Se; silicon, Si; *etc.*) and nutrients (nitrogen, N; phosphorus, P; potassium, K, calcium, Ca; *etc.*) have been found effective in mitigating HT stress-induced damage in plants [15–21].

Development of new crop cultivars tolerant to HT is a major challenge for plant scientists [13,22]. Depending upon the extremity and duration, and also depending upon the plant types and other environmental factors in the surroundings, plants show dynamic responses to HT, but identification and confirmation of the traits that confer tolerance to HT still remain elusive [23,24]. Plant scientists involved in research on HT stress are endeavoring to discover the plant responses that lead to heat tolerance and they are also trying to investigate how plants can be managed in HT environments. Recent widely studied molecular approaches have included omics techniques and the development of transgenic plants through manipulation of target genes [25–27]. Investigation of these underlying molecular processes may provide ways to develop stress tolerant varieties and to grow agriculturally important crop plants under HT. In this chapter, we focus on these new strategies and we review the recent research into the physiological and biochemical events and the molecular responses seen in plants in response to HT stress. We also review the roles of exogenous protectants, the underlying mechanisms for transduction of HT stress signals, and transgenic approaches currently being taken to promote HT stress tolerance in plants.

## 2. Plant Response to Heat Stress

Plant responses to HT vary with the degree of temperature, duration and plant type. At extreme HT, cellular damage or cell death may occur within minutes, which may lead to a catastrophic collapse of cellular organization [28]. Heat stress affects all aspects of plant processes like germination, growth, development, reproduction and yield [5,29–31]. Heat stress differentially affects the stability of various proteins, membranes, RNA species and cytoskeleton structures, and alters the efficiency of enzymatic reactions in the cell for which the major physiological processes obstacle and creates metabolic imbalance [32–35]. Some common effects of heat stress have been summarized in Table 1.

### 2.1. Growth

Among the growth stages of plant the germination is affected first of all. Heat stress exerts negative impacts on various crops during seed germination though the ranges of temperatures vary largely on crop species [49,50]. Reduced germination percentage, plant emergence, abnormal seedlings, poor seedling vigor, reduced radicle and plumule growth of geminated seedlings are major impacts caused by heat stress documented in various cultivated plant species [50–52]. Inhibition of seed germination is also well documented in HT which often occurs through induction of ABA [53]. At very HT (45 °C) the rate of germination of wheat was strictly prohibited and caused cell death and embryos for which seedling establishment rate was also reduced [54]. Plant height, number of tillers and total biomass were reduced in rice cultivar in response to HT [55].

High temperature causes loss of cell water content for which the cell size and ultimately the growth is reduced [24,56]. Reduction in net assimilation rate (NAR) is also another reason for reduced relative growth rate (RGR) under HT which was confirmed in maize and millet [57] and sugarcane [58]. The morphological symptoms of heat stress include scorching and sunburns of leaves and twigs, branches and stems, leaf senescence and abscission, shoot and root growth inhibition, fruit discoloration and damage [24]. Damage to leaf-tip and margins, and rolling and drying of leaves, necrosis, was observed in sugarcane due to HT stress [59]. In common bean (*Phaseolus vulgaris*) morphophysiological characteristics such as phenology, partitioning, plant-water relations, and shoot growth and extension are seriously hampered by heat stress [60]. In some plant species growth at HTs (28/29 °C) cause noteworthy elongated stems and entended leaves (hyponasty) and diminish in total biomass [61,62]. Reduced number of tillers with promoted shoot elongation was observed in wheat plant under heat stress [50]. In wheat green leaf area and productive tillers/plant were drastically reduced under HT (30/25 °C, day/night) [39]. High temperatures may alter the total phenological duration by reducing the life period. Increases in temperatures 1–2 °C than the optimum result in shorter grain filling periods and negatively affect yield components of cereal [22,63]. In *T. aestivum* HT (28 °C to 30 °C) reduced the germination period, days to anthesis booting, maturity that is ultimate the total growth duration [64]. At extreme heat stress plants can show programmed cell death in specific cells or tissues may occur within minutes or even seconds due to denaturation or aggregation of proteins, on the other hand moderately HTs for extended period cause gradual death; both types of injuries or death can lead to the shedding of leaves, abortion of flower and fruit, or even death of the entire plant [14,24].

### 2.2. Photosynthesis

Photosynthesis is one of the most heat sensitive physiological processes in plants [65]. High temperature has a greater influence on the photosynthetic capacity of plants especially of C_3_ plants than C_4_ plants [66]. In chloroplast, carbon metabolism of the stroma and photochemical reactions in thylakoid lamellae are considered as the primary sites of injury at HTs [67,68]. Thylakoid membrane is highly susceptible to HT. Major alterations occur in chloroplasts like altered structural organization of thylakoids, loss of grana stacking and swelling of grana under heat stress [24,56]. Again, the photosystem II (PSII) activity is greatly reduced or even stops under HTs [69]. Heat shock reduces the amount of photosynthetic pigments [68].

The ability of plant to sustain leaf gas exchange and CO_2_ assimilation rates under heat stress is directly correlated with heat tolerance [66,70]. Heat markedly affects the leaf water status, leaf stomatal conductance (*gs*) and intercellular CO_2_ concentration [71]. Closure of stomata under HT is another reason for impaired photosynthesis that affects the intercellular CO_2_ [56]. The decline in chl pigment also is a result of lipid peroxidation of chloroplast and thylakoid membranes as observed in sorghum due to heat stress (40/30 °C, day/night) [40]. Photosystem II photochemistry (Fv/Fm ratio) and *gs* were also reduced under the same stress condition. All these events significantly decreased the photosynthesis compared with OT in sorghum [40]. In soybean, heat stress (38/28 °C) significantly decreased total chl content (18%), chl *a* content (7%), chl *a*/*b* ratio (3%), Fv/Fm ratio (5%), Pn (20%) and *gs* (16%). As a result decreased in sucrose content (9%) and increased reducing sugar content (47%) and leaf soluble sugars content (36%) were observed [44]. In rice plants, HT (33 °C, 5 days) decreased the photosynthetic rate by 16% in the variety Shuanggui 1 and 15% in T219 [37]. Greer and Weedon [72] observed that average rates of photosynthesis of *Vitis vinifera* leaves decreased by 60% with increasing temperature from 25 to 45 °C. This reduction in photosynthesis was attributed to 15%–30% stomatal closure.

Some other reasons believed to hamper photosynthesis under heat stress are reduction of soluble proteins, Rubisco binding proteins (RBP), large-subunits (LS), and small-subunits (SS) of Rubisco in darkness, and increases of those in light [73]. High temperature also greatly affects starch and sucrose synthesis, by reduced activity of sucrose phosphate synthase, ADP-glucose pyrophosphorylase, and invertase [24,74]. Heat imposes negative impacts on leaf of plant like reduced leaf water potential, reduced leaf area and pre-mature leaf senescence which have negative impacts on total photosynthesis performance of plant [71,75]. Under prolonged heat stress depletion of carbohydrate reserves and plant starvation are also observed [74].

### 2.3. Reproductive Development

Although all plant tissues are susceptible to heat stress at almost all the gowth and developmental stages, the reproductive tissues are the most sensitive, and a few degrees elevation in temperature during flowering time can result in the loss of entire grain crop cycles [30]. During reproduction, a short period of heat stress can cause significant decrease in floral buds and flowers abortion although great variations in sensitivity within and among plant species and variety exists [76]. Even heat spell at reproductive developmental stages plant may produces no flowers or flowers may not produce fruit or seed [77,78]. The reasons for increasing sterility under abiotic stress conditions including the HT are impaired meiosis in both male and female organs, impaired pollen germination and pollen tube growth, reduced ovule viability, anomaly in stigmatic and style positions, reduced number of pollen grains retained by the stigma, disturbed fertilization processes, obstacle in growth of the endosperm, proembryo and unfertilized embryo [79].

HT treatment (>33 °C) at heading stage significantly reduced anther dehiscence and pollen fertility rate, leading to reduction in the number of pollens on the stigma which were the causes of reduced fertilization and subsequent spikelet fertility and sterile seed in rice [37,80] where the sensitive varieties were more suseptable to this occurrence compared to the tolerant varieties [37]. High night temperatures (32 °C) increase in spikelet sterility (by 61% compared to control) in rice which was resulted from decreased pollen germination (36%) of rice [41]. High temperature often causes excessive ethylene (Eth) production and leads to male sterility of rice pollens. The Eth is hypothesized to inhibit the key enzymes in sugar–starch metabolism which weaken sink strength and restrict grain filling and ultimately produce sterile grain. Due to late sowing-induced heat stress the ear length, number of spikelet main stem^−1^, no. of fertile floret main stem^−1^ were reduced significantly in wheat plant those resulted in reduced grain yield [81]. Edreira and Otegui [47] observed that heat stress at flowering periods, more specifically at pre-silking and silking stages resulted higher yield reduction relative to the heat stress at grain filling stage of maize. High temperature stress resulted in abscission and abortion of flowers, young pods and developing seeds, resulting in lower seed numbers in soybean [82]. High temperatures at flowering are known to decrease pollen viability in soybean [44].

### 2.4. Yield

Elevated temperatures are raising apprehension regarding crop productivity and food security [62]. Its affect is so terrible that even a small (1.5 °C) increase in temperature have significant negative effects on crop yields [83]. Higher temperatures affect the grain yield mostly through affecting phenological development processes. Heat induced yield reduction was documented in many cultivated crops including cereals (e.g., rice, wheat, barley, sorghum, maize), pulse (e.g., chickpea, cowpea), oil yielding crops (mustard, canola) and so on [47,78,80,82,84,85].

It was demonstrated that increase of the seasonal average temperature 1 °C decreased the grain yield of cereals by 4.1% to 10.0% [86]. The sensitive crop varieties are more severely affected by heat stress relative to tolerant varieties. At heat stress of 35–40 °C the 1000-grain weight was reduced by 7.0%–7.9% in sensitive Shuanggui 1 and 3.4%–4.4% in tolerant Huanghuazhan variety of rice. The higher yield reduction was also observed in heat-sensitive rice cultivar Shuanggui 1 (35.3% to 39.5%) compared to heat tolerant cultivar Huanghuazhan (21.7% to 24.5%) [80]. High night temperature (32 °C) decreased grain length (2%), width (2%), and weight in *O. sativa* and increased spikelet sterility (61%). It also increased grain nitrogen (N) concentration (44%) which was inversely related to grain weight. All of these factors contributed to reduced yield (90%) [41]. Heat stress modifies the early dough and maturity stage shorten the kernel desiccation period and cause grain yield loss in wheat [48]. Heat also reduces the single kernel weight and it is the major contributor to the yield loss [87]. Compared to OT late sowing mediated heat stress (28–30 °C) caused significant reduction of yield in different wheat varieties, viz. 70% reduction in “Sourav”, 58% in “Pradip”, 73% in “Sufi”, 55% in “Shatabdi” and 53% in “Bijoy” [64]. In sorghum, due to heat stress, filled seed weight and seed size were reduced by 53% and 51% respectively, which ultimately reduced the yield [40]. In canola (*Brassica* spp.), seed yield on the main stem was reduced by 89%, but all branches contributed to overall yield loss of 52% at HT of over 30 °C. The cause of this yield decline was due to heat induced infertile pods, reduced seed weight and seeds per pod [88].

Loss of productivity in heat stress is chiefly related to decreased assimilatory capacity [89] which is due to reduced photosynthesis by altered membrane stability [22] and enhanced maintenance respiration costs [90], reduction in radiation use efficiency (RUE, biomass production per unit of light intercepted by the canopy). These occurrences were documented in wheat [91] and maize [92]. High temperature (33–40 °C) in maize negatively affected light capture, RUE, biomass and gain yield, harvest index although heat at the flowering stage resulted higher yield reduction than at grain filling period [47]. Elevated temperature affects the performance and crop quality characteristics. Grain quality characteristics in barley significantly changed under heat stress. In barley grain several proteinogenic amino acids concentrations and maltose content increased, where the concentrations of total non-structural carbohydrates, starch, fructose and raffinose, lipids and aluminum were reduced [93]. Damages in pod quality parameters such as fibre content and break down of the Ca pectate were found in okra (*Abelmoschus esculentus*) at HT stress [46].

### 2.5. Oxidative Stress

**Figure 2:**
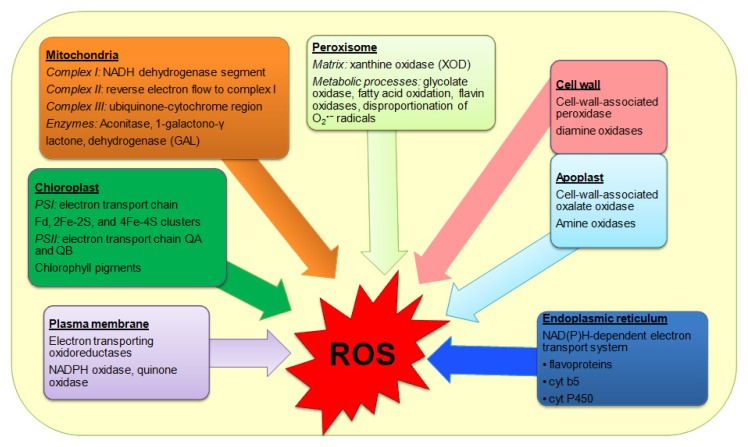
Sites of production of reactive oxygen species in plants [5].

Different metabolic pathways are depended upon enzymes which are sensitive to various degrees of HTs. It has been suggested that, like other abiotic stress, heat stress might uncouple enzymes and metabolic pathways which cause the accumulation of unwanted and harmful ROS most commonly singlet oxygen (^1^O_2_), superoxide radical (O_2_^·−^), hydrogen peroxide (H_2_O_2_) and hydroxyl radical (OH^·^) which are responsible for oxidative stress [94]. The reaction centers of PSI and PSII in chloroplasts are the major sites of ROS generation though ROS are also generated in other organelles viz. peroxisomes and mitochondria [95]. A linear relationship exists between maximal efficiency of PSII and the accumulated ROS. It is suggested that because of thermal damage to photosystems under such HTs less absorbtion of photon occurs [96]. In such stress conditions, if photon intensity is absorbed by PSI and PSII, the excess of which is required for CO_2_ assimilation are considered as surplus electrons, those serve as the source of ROS [96]. Among the ROS, O_2_^·−^ is formed by photooxidation reactions (flavoprotein, redox cycling), through Mehler reaction in chloroplasts, during mitochondrial ETCs reactions and glyoxisomal photo respiration, by NADPH oxidase in plasma membranes, xanthine oxidase and membrane polypeptides (Figure 2). Hydroxyl radical is formed due to the reaction of H_2_O_2_ with O_2_^·−^ (Haber- Weiss reaction), reactions of H_2_O_2_ with Fe^2+^ (Fenton reaction) and decomposition of O_3_ in apoplastic space [97,98] (Figure 2). Singlet oxygen is formed during photoinhibition, and PS II electron transfer reactions in chloroplasts [99,100]. Hydroxyl radical is not considered to have signaling function although the products of its reactions can elicit signaling responses, and cells sequester the catalytic metals to metallochaperones efficiently avoiding OH^·^ [97,98].

Various physiological damages occur in plants upon exposures to varying levels of heat stress [96]. Hydroxyl radicals can potentially react with all biomolecules, like pigments, proteins, lipids and DNA, and almost with all constituents of cells [97,98]. Singlet oxygen can directly oxidize protein, polyunsaturated fatty acids and DNA [99,100]. Thermal stress can induce oxidative stress through peroxidation of membrane lipids and disruption of cell membrane stability by protein denaturation [24,101]. Functional decrease in photosynthetic light reaction even under moderate HTs was documented to induce oxidative stress through ROS production caused by increased electron leakage from the thylakoid membrane [96,102]. The HT increased leaf temperature which reduced the antioxidant enzyme activities that increased malondialdehyde (MDA) content in leaves of rice plant [37]. Heat stress (33 °C) induced oxidative stress was observed to damage membrane properties, protein degradation, enzyme deactivation in wheat that reduced the cell viability remarkably. Heat stress induced oxidative stress also significantly increased the membrane peroxidation and reduced the membrane thermostability by 28% and 54% which surprisingly increased electrolyte leakage in wheat [103]. Populations of perennial ryegrass (*Lolium perenne* L.) when were exposed to moderate (36 °C) and severe HT stress (40 °C), oxidative stress was prominent which was proved by the presence of higher H_2_O_2_ level, and it was responsible for remarkable physiological damage of maximal efficiency of PS II, destroyed cell membrane stability and caused lipid peroxidation [96]. High temperature stress provoked membrane lipid peroxidation and aggravated membrane injury was also observed in cotton, sorghum and soybean [40,44,75]. In sorghum relative to control heat stress (40/30 °C, day/night) increased membrane damage and MDA content by 110% and 75%, respectively which was accompanied by increased H_2_O_2_ and O_2_^·−^ content (124% and 43%, respectively) [40]. Moreover, the ROS produced by HT stress are involved in proteolysis of protein or degradation of polymeric protein into simple soluble forms those are the cause of premature leaf senescence in cotton [75]. In wheat 2 days of heat exposure resulted root growth inhibition which was correlated with powerful oxidative stress as evidenced by a significant increase (68%) of O_2_^·−^ production in root cells. The MDA content also increased by 27% in the first leaf 2 days after exposure at the early stages of seedling development, and this trend also continued during the later stages of development (by 58%) [104].

Continual heat stress causes the ROS accumulation at the plasma membrane outer surface which can cause membrane depolarization [105], activation of Ca-induced RBOHD (the ROS-producing enzyme RBOHD located at the plasma membrane). In such extreme cases, ROS accumulation in cells can trigger programmed cell death [105]. Although the ROS have tremendous destructive effects on plant metabolic processes they have also hypothesized to have signaling behaviors to trigger the heat shock responses towards the development of heat tolerance in plant which are inexplicable and should be divulged [94].

## 3. Plant Adaptation to Heat Stress

**Figure 3:**
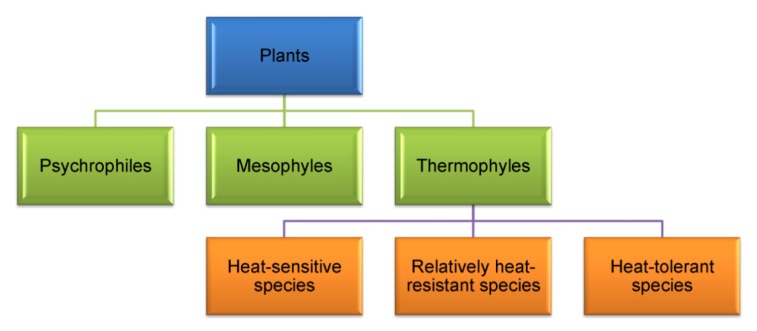
Classification of plants on the basis of their heat tolerance.

Living organisms can be classified into three groups, subject to the preferred temperature of growth (Figure 3). There are (a) Psychrophiles: which grow optimally at low temperature ranges between 0 and 10 °C; (b) Mesophyles: which favor moderate temperature and grow well between 10 and 30 °C; and (c) Thermophyles: which grow well between 30 and 65 °C or even higher [106]. There is a great variation among the plant species in terms of their response and tolerance to HT. On the basis of thermotolerance, Larcher [107] classified all the plant species into three groups (Figure 3).

**Figure 4:**
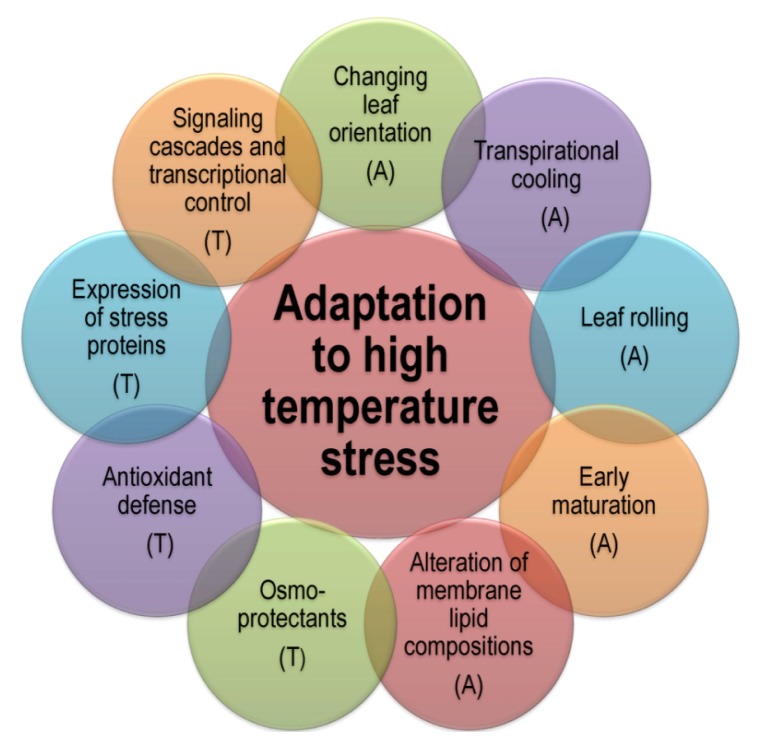
Different adaptation mechanisms of plants to high temperature. A: Avoidance, T: Tolerance.

Survival in hot, dry environments can be achieved in a variety of ways, by combinations of adaptations [108]. Plant adaptation to heat stress includes avoidance and tolerance mechanisms which employ a number of strategies (Figure 4).

### 3.1. Avoidance Mechanisms

Under HT conditions, plants exhibit various mechanisms for surviving which include long-term evolutionary phenological and morphological adaptations and short-term avoidance or acclimation mechanisms such as changing leaf orientation, transpirational cooling, or alteration of membrane lipid compositions. Closure of stomata and reduced water loss, increased stomatal and trichomatous densities, and larger xylem vessels are common heat induced features in plant [58]. In many crop plants, early maturation is closely correlated with smaller yield losses under HT, which may be attributed to the engagement of an escape mechanism [24,109]. Plants growing in a hot climate avoid heat stress by reducing the absorption of solar radiation. This ability is supported by the presence of small hairs (tomentose) that form a thick coat on the surface of the leaf as well as cuticles, protective waxy covering. In such plants, leaf blades often turn away from light and orient themselves parallel to sun rays (paraheliotropism). Solar radiation may also be reduced by rolling leaf blades. Plants with small leaves are also more likely to avoid heat stress: they evacuate heat to ambient more quickly due to smaller resistance of the air boundary layer in comparison with large leaves. Plants rely on the same anatomical and physiological adaptive mechanisms those are deployed in a water deficit to limit transpiration. In well-hydrated plants, intensive transpiration prevents leaves from heat stress, and leaf temperature may be 6 °C or even 10–15 °C lower than ambient temperature. Many species have evolved life histories which permit them to avoid the hottest period of the year. This can be achieved by leaf abscission, leaving heat resistant buds, or in desert annuals, by completing the entire reproductive cycle during the cooler months [108]. Such morphological and phenological adaptations are commonly associated with biochemical adaptations favoring net photosynthesis at HT (in particular C_4_ and CAM photosynthetic pathways), although C_3_ plants are also common in desert floras [108]. High temperature can affect the degree of leaf rolling in many plants. Physiological role of leaf rolling was the maintenance of adaptation potential by increasing the efficiency of water metabolism in the flag leaves of wheat under HT [110]. During active growth, all plants are highly sensitive to temperature stress. Selected species of land plants increase their resistance to heat only in the summer, while others demonstrate the highest level of tolerance during winter dormancy. Dormant plants become resistant to stress upon reaching a developmental stage induced by factors other than high environmental temperature. In many land plant species, noticeable changes in heat tolerance are not observed. Due to the close correlation between drought and HT, the effects of each stressor on field-grown plants can be difficult to distinguish, and adaptations to arid environments can be effective only if they lead to avoidance or tolerance of both stresses [108].

High temperature stress can also be avoided by crop management practices such as selecting proper sowing methods, choice of sowing date, cultivars, irrigation methods, *etc.* For instance, in subtropical zones, cool-season annuals such as lettuce when sown in the late summer may show incomplete germination and emergence due to high soil temperature [111]. The incomplete emergence problem can be overcome by sowing the lettuce seed into dry beds during the day and then sprinkle irrigating the beds during the late afternoon. Seed priming is another potential solution to this problem which involves placing the seed in an osmotic solution for several days at moderate temperatures and then drying them. In contrast, tropical crops may face inadequate plant emergence and establishment can limit the productivity of several warm-season annual crops due to very hot soil surface. In such cases, deep placement can overcome the problem. In temperate or subtropical climatic zones, which have seasonal variations in temperature, sowing date can be varied to increase the probability that annual crop species will escape stressfully HTs during subsequent sensitive stages of development. In some cases, HT and intense direct solar radiation can cause damage to fruit. This can be avoided if fruit is shaded by foliage [111].

### 3.2. Tolerance Mechanisms

Heat tolerance is generally defined as the ability of the plant to grow and produce economic yield under HT. This is a highly specific trait, and closely related species, even different organs and tissues of the same plant, may vary significantly in this respect. Plants have evolved various mechanisms for thriving under higher prevailing temperatures. They include short term avoidance/acclimation mechanism or long term evolutionary adaptations. Some major tolerance mechanisms, including ion transporters, late embryogenesis abundant (LEA) proteins, osmoprotectants, antioxidant defense, and factors involved in signaling cascades and transcriptional control are essentially significant to counteract the stress effects [24,112].

In case of sudden heat stress, short term response, *i.e.*, leaf orientation, transpirational cooling and changes in membrane lipid composition are more important for survival [24,113]. Smaller yield losses due to early maturation in summer shows possible involvement of an escape mechanism in heat stress tolerance [109]. Different tissues in plants show variations in terms of developmental complexity, exposure and responses towards the prevailing or applied stress types [114]. The stress responsive mechanism is established by an initial stress signal that may be in the form of ionic and osmotic effect or changes in the membrane fluidity. This helps to reestablish homeostasis and to protect and repair damaged proteins and membranes [115].

## 4. Antioxidant Defense in Response to Heat-Induced Oxidative Stress

Plants must be protected from heat-induced oxidative stress so that they can survive under HT. Tolerance to HT stress in crop plants has been associated with an increase in antioxidative capacity [116,117]. Studies on heat-acclimated *versus* non-acclimated cool season turfgrass species suggested that the former had lower production of ROS as a result of enhanced synthesis of ascorbate (AsA) and glutathione (GSH, [118]). Available data suggest that some signaling molecules may cause an increase in the antioxidant capacity of cells [119,120].

Tolerant plants entail a tendency of protection against the damaging effects of ROS with the synthesis of various enzymatic and nonenzymatic ROS scavenging and detoxification systems [121]. Activities of different antioxidant enzymes are temperature sensitive and activation occurs at different temperature ranges but the activities of these enzymes increase with increasing temperature. Chakrabortty and Pradhan [122] observed that catalase (CAT), ascorbate peroxidase (APX) and superoxide dismutase (SOD) showed an initial increase before declining at 50 °C, while peroxidase (POX) and glutathione reductase (GR) activities declined at all temperatures ranging from 20 to 50 °C. In addition, total antioxidant activity was at a maximum at 35–40 °C in the tolerant varieties and at 30 °C in the susceptible ones. Their activities also differ depending upon tolerance or susceptibility of different crop varieties, their growth stages and growing season [116,122].

Antioxidant metabolites like AsA, GSH, tocopherol and carotene also protect plants against oxidative stress [123]. Heat acclimated turf grass showed lower production of ROS as a result of enhanced synthesis of AsA and GSH [118]. In wheat, it was established that heat stress induced accumulation of GSH levels and increased the activity of the enzymes involved in GSH synthesis and the GSH/GSSG ratio [124]. In fact, heat stress increased GSH levels in the flag leaf of two wheat genotypes with contrasting behavior in heat tolerance at all the stages during grain development [125]. Balla *et al.* [126] demonstrated the importance of the antioxidant enzyme system in defense against heat stress. The activity of the enzymes glutathione *S*-transferase (GST), APX and CAT was more enhanced in the cultivar showed better tolerance to heat stress and projection against ROS production. They reported that the tolerance of the wheat varieties appeared to be correlated with the antioxidant level, though changes in activity were observed for different antioxidant enzymes. Almeselmani *et al.* [116] concluded that various antioxidant enzymes showed positive correlation with chl content and negative with membrane injury index at most of the stages in the five wheat genotypes. Later, they [127] reported that the antioxidant defense mechanism plays an important role in the heat stress tolerance of wheat genotypes and it was observed that the activities of SOD, APX, CAT, GR and POX increased significantly at all stages of growth in heat tolerant cultivers (C 306) in response to heat stress treatment, while susceptible cultivar (PBW 343) showed a significant reduction in CAT, GR and POX activities in the HT treatment.

Rani *et al.* [128] exposed 5-d-old thermo tolerant genotype, namely BPR-542-6, and thermo susceptible genotype, namely NPJ-119, of *B. juncea* to HT (45.0 ± 0.5 °C) stress. They observed that the activities of SOD, POX, CAT, APX and GR increased under HT stress but the increase was significantly higher in tolerant genotype. The basal level of all antioxidative enzymes except CAT was found more in tolerant genotype. On revival, SOD and CAT started decreasing but activity of POX and GR still continued increasing in both the genotypes however, APX showed differential behavior on revival, it continued increasing in tolerant genotype and started decreasing in susceptible genotype. Recently, Kumar *et al.* [129] studied the comparative response response of HT in *O. sativa* and *Z. mays* plants and also investigated the antioxidant defense system under stress. They observed that the expression of enzymatic antioxidants like CAT, APX and GR was found to be higher in *Z. mays* plants compared to *O. sativa* plants while no variations existed for superoxide dismutase at 45/40 °C. In addition, the non-enzymatic antioxidants like AsA and GSH were maintained significantly greater levels at 45/40 °C in maize than in *O. sativa* genotypes. These findings suggested that *Z. mays* genotypes were able to retain their growth under HT partly due to their superior ability to cope up with oxidative damage by heat stress compared to *O. sativa* genotypes. Since, *Z. mays* and *O. sativa* belong to C_4_ and C_3_ plant groups, respectively, these observations may also reflect the relative sensitivity of these plant groups to heat stress. While studying with *T. aestivum* seedlings, Hasanuzzaman *et al.* [18] observed that heat treatment (38 °C, 24 and 48 h) resulted in an increase of the activities of antioxidant enzymes—APX, GR, GPX and GST. However, supplementation of heat-treated seedlings with SNP (NO donor) significantly upregulated the activities of antioxidant enzymes and protected wheat seedlings from HT induced oxidative stress. Generally, an increase in temperature leads to an increased expression of the antioxidative enzymes until a particular temperature after which they decline. The temperature until which increased activities are maintained varies in the tolerant and susceptible varieties. In the tolerant varieties, they could maintain increased activities at HT in comparison to the susceptible ones [122].

## 5. Mechanism of Signal Transduction and Development of Heat Tolerance

**Figure 5:**
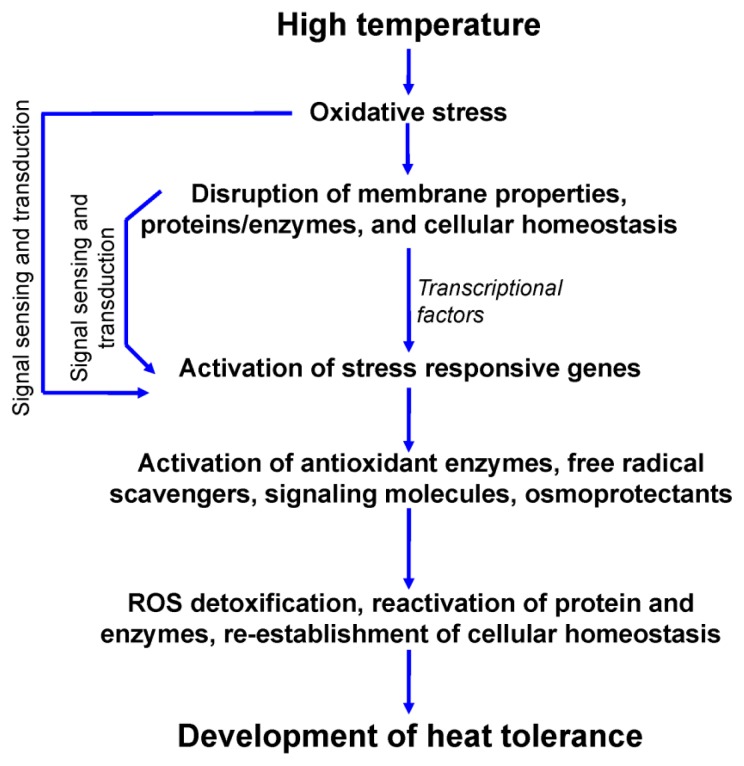
Schematic illustration of heat induced signal transduction mechanism and development of heat tolerance in plants.

Upregulation of many genes has been reported to help the plant to withstand the stress conditions which leads to plant adaptation [130]. Upon stress plants perceive the external and internal signals through different independent or interlinked pathways which are used to regulate various responses for its tolerance development ([131]; Figure 5). Plant responses to stress are complex integrated circuits within which multiple pathways are involved. To generate response in specific cellular compartments or tissues against a certain stimuli, interaction of cofactors and signaling molecules are required. Signaling molecules are involved in activation of stress responsive genes. There are various signal transduction molecules related to stress responsive gene activation depending upon plant type, types of stresses. Some broad group of those are the Ca-dependent protein kinases (CDPKs), mitogen-activated protein kinase (MAPK/MPKs), NO, sugar (as signaling molecule), phytohormones [132]. These molecules together with transcriptional factors activate stress responsive genes.

Once the stress responsive genes activate, these help to detoxify the ROS (by activating detoxifying enzymes, free radical scavengers); reactivate the essential enzymes and structural proteins [133] and all the above stated processes help to maintain the cellular homeostasis (Figure 5). This can be said as a typical model through which heat resistance or tolerance developed within the plant. But to understand the signaling molecules and pathways involved in heat tolerance development intrinsic research is required indeed. Based on several recent papers Proveniers and van Zanten [134] described the underlying signaling mechanisms under HT stress which was mostly found to involve the basic helix-loop-helix (bHLH) transcription factor phytochrome interacting factor 4 (PIF4) whose orthologs have been identified in several crop species. The PIF4 controls acclimation to changes in ambient temperature which can, therefore, be considered an important contributor to the competitive ability in natural populations and feeds directly into important hormonal and developmental pathways tuning the acclimation mechanisms. For instance, PIF4 alleles has been shown to contribute to early inflorescence internode elongation, probably via control of floral timing which may be used for targeted breeding approaches to improve tolerance to HT.

## 6. Use of Exogenous Protectants in Mitigating Heat-Induced Damages

One of the ways to deal with adverse effects of heat stress may involve exploring some molecules that have the potential to protect the plants from the harmful effects of HT. In recent decades, exogenous application of protectant such as osmoprotectants, phytohormones, signaling molecules, trace elements, *etc.*, have shown beneficial effect on plants grown under HT as these protectants has growth promoting and antioxidant capacity [18,135–137]. Some of their effects are summarized in Table 2.

Accumulation of osmolytes such as Pro, GB and Tre is a well-known adaptive mechanism in plants against abiotic stress conditions including HT. Since heat sensitive plants apparently lack the ability to accumulate these substances, heat tolerance in such plants can be improved by exogenous application of osmoprotectants [138,153–155]. Rasheed *et al.* [138] showed that soaking sugarcane nodal buds in exogenous Pro (20 mM) and GB (20 mM) solutions exhibited some positive changes in some physiological and anatomical characteristics and possibility of mitigating the adversities of heat stress. Supplementation with Pro and GB considerably reduced the H_2_O_2_ production, improved the accumulation of soluble sugars and protected the developing tissues from heat stress effects. However, Pro was more effective than GB in that study. Exogenous Pro and GB application also improved the K^+^ and Ca^2+^ contents, and increased the concentrations of free Pro, GB and soluble sugars which rendered the buds more tolerant to HT. Kaushal *et al.* [139] reported that exogenous Pro ensured protection of vital enzymes of carbon and antioxidant metabolism which might be the basis of heat tolerance in chickpea (*Cicer arietinum* L.) plants. They observed that exogenous application of Pro in heat stressed (45/40 °C) plants showed less injury to membranes, had improved water and chl content. The plants supplemented with Pro also significantly reduced oxidative injury coupled due to enhanced levels of enzymatic and non-enzymatic antioxidants. With Pro application, the MDA and H_2_O_2_ content in shoots decreased by 32% and 20%, respectively compared to those growing without Pro at same temperatures. A significant improvement was also noticed in the activities of enzymes of carbon metabolism in Pro-treated plants. These results suggest that exogenous Pro imparts partial heat tolerance to chickpea [139]. In a recent study, Kumar *et al.* [140] found that exogenous application of Pro, GB and Tre (10 μM) promoted the growth in heat-stressed chickpea plants.

Exogenous applications of several phytohormones were found to be effective in mitigating heat stress in plants. Chhabra *et al.* [143] studied the phtohormones induced amelioration of HT stress in *Brassica juncea* and found that soaking seeds in 100 μM IAA, 100 μM GA, 50 and 100 μM Kinetin and 0.5 & 1 μM ABA were effective for mitigating the effect of heat stress (47 ± 0.5 °C). The significant observation was that both growth promoting and growth retarding hormones were effective in mitigation of heat stress effects. The role of growth promoting hormone in the mitigation of heat stress was at a concentration which was otherwise lethal or toxic to its growth seedling stage. Salicylic acid is a plant hormone found to be an effective protectant under heat stress. Wang and Li [156] reported that spraying with a 0.1 mM SA decreased thiobarbituric acid reactive substances (TBARS) and relative electrolyte leakage in young grape leaves under heat stress, indicating that SA can induce intrinsic heat tolerance in grapevines. In grapevine leaves, SA pretreatment alleviated the heat stress induced decrease in Pn mainly through maintaining higher Rubisco activition state, and it accelerated the recovery of Pn mainly through its effects on PSII function [142]. These effects of SA were thought to be related in part to enhanced levels of heat shock protein 21 (HSP21). However, SA did not influence the Pn of leaves before heat stress. In rice, pretreatment of the seedlings with SA (0.5 mM) retarded the heat stress (35 °C, 48 h)-induced electrolyte osmosis, reduced MDA content and superoxide anion radical (O_2_^·−^) production rate. In contrast, the contents of H_2_O_2_, Pro, soluble sugar, soluble protein, AsA and GSH in rice seedlings increase with SA pretreatment under HT stress. These findings suggested that SA pretreatment enhanced the heat tolerance of rice seedling. Although relatively less reports are available about metabolic mechanisms through which ABA acts in inducing heat tolerance. Some earlier reports indicate that exogenous ABA application confers heat tolerance in crop [157]. Abscisic acid itself a signaling molecule, but sometimes ABA may impart thermotolerance by raising the levels of other signaling molecules like NO. Kumar *et al.* [140] investigated the interactive effects of ABA and osmolytes in chickpea plants and they reported that exogenous application of ABA (2.5 μM) significantly mitigated the seedling growth at 40/35 and 45/40 °C. Exogenous ABA also facilitated the increase in growth which was associated with enhancement of endogenous levels of ABA and osmolytes. The oxidative damages in ABA treated plants were also much lower than non-treated plants under heat stress condition which was indicated by reduced MDA and H_2_O_2_ contents. In the contrary, inhibitor of ABA biosynthesis, fluridone (FLU) reverted the actions induced by ABA which suggest a clear role of ABA in mitigating heat-induced damages. Chen *et al.* [146] treated grape seedlings with 50 μM JA solution and observed that that JA could extenuate the change of stress under heat stress (42 °C). This protection was accompanied by the upregulation of antioxidant enzymes’ (SOD, CAT and POD) activity compared with these untreated under heat stress. Kumar *et al.* [145] investigated the effect of different concentrations of 24-epibrassinolide (24-EBL) on growth, antioxidant enzyme of mustard (*B. juncea*) seedlings. Results showed that the seedlings which were treated with different concentrations of 24-EBL showed better growth and enhanced protein content under heat stress. Exogenous 24-EBL also upregulated the activities of antioxidant enzymes like SOD, CAT, POX which rendered the plants more tolerant to heat-induced oxidative stress. The protective effects of BRs were also observed in bean plants subjected to heat stress. In an experiment El-Bassiony *et al.* [144] sprayed bean plants with different concentrations of BRs (25, 50 and 100 mg L^−1^). They observed that spraying bean plants with BRs at a concentration of 25 and 50 mg L^−1^ increased vegetative growth, total yield and quality of pods under HT. However, there was no difference between the treatments. Spraying of 25 mg L^−1^ BR increased the total free amino acids (FAA) in leaves and total phenolic acids in the pod compared to control.

Nitric oxide is a signaling molecule involved in many physiological processes in plants and it also acts a vital role in plants tolerance to abiotic stress including HT [15,18,20,135]. Song *et al.* [147] pretreated callus of *Phragmites communis* (reed) with two different NO donors viz. SNP and *S*-nitroso-*N*-acetylpenicillamine (SNAP) for 24 h and then exposed to HT (45 °C) for 2 h. They observed that exogenous NO caused dramatic alleviation of HT induced ion leakage increase, growth suppression and cell viability as well as H_2_O_2_ and MDA contents. However, the activities of SOD, CAT, APX and POD increased in both calluses in the presence of NO donors under heat stress. On the other hand, NO scavenger (cPTIO) arrested NO donors mediated protective effects. They provided a good indication that NO can effectively overcome oxidative stress induced by heat stress and that NO might act as a signal in activating ROS scavenging enzymes under heat stress and thus confer thermotolerance [147]. In a recent study, it was reported that excessive NO production under HT might be involved in the thermoinhibition of seed germination in *Arabidopsis thaliana* [158]. In our recent study, we investigated the protective role of exogenous NO in alleviating HT induced damages of wheat (*Triticum aestivum* L. cv. Pradip) seedlings [20]. Heat treatment (38 °C) alone or in combination with 0.5 mM SNP (a NO donor) was applied with nutrient solution on 8-d-old hydroponically grown seedlings for a period of 24 and 48 h. Heat stress significantly decreased the chl content and increased the MDA and H_2_O_2_ levels in time depending manners. Ascorbate (AsA) content markedly decreased upon heat treatment but GSH and glutathione disulfide (GSSG) content increased. Heat treatment resulted in an increase of the activities of antioxidant enzymes—APX, GR, glutathione peroxidase (GPX) and GST. The activities of glyoxalase enzymes also increased upon heat stress. Exogenous NO supplementation in the seedlings grown under OT had little influence on the nonenzymatic and enzymatic components compared to the control. However, supplementation of heat-treated seedlings with SNP significantly reduced the HT induced lipid peroxidation, H_2_O_2_ content and increased the content of chl, AsA and GSH as well as the GSH/GSSG ratio. Heat treated seedlings which were supplemented with SNP also upregulated the activities of APX, monodehydroascorbate reductase (MDHAR), dehydroascorbate reductase (DHAR), GR, GST, CAT and glyoxalase I (Gly I). Our results suggested that exogenous supply of NO protects wheat seedlings from HT induced oxidative stress by upregulating antioxidant defense and glyoxalase system [18].

Polyamines (PAs) are low molecular mass aliphatic amines and organic polycations found in a wide range of organisms from bacteria to plants and animals [159]. They also play important roles in plants’ responses to abiotic stress. A large amount of data exists demonstrating that an accumulation of the three main PAs occurs under many types of abiotic stresses [160]. Polyamine provides protection to plant from HT stress in different ways. They can affect photosynthesis in different ways. Structure and function of the photosynthetic apparatus can be regulated effectively by PAs. Polyamines are able to maintain thermostability of thylakoid membranes under heat thus increase photosynthetic efficiency [55,161]. In case of wheat delaying the date of sowing resulted in HT stress in wheat plant and caused a marked reduction in the growth, duration of plant from sowing to maturity by about 30 day. Yield and its components like number of spikes per plant; weight of grains per plant; weight of 1000- grains; straw yield per plant and crop and harvest index were also significantly reduced as compared with sowing at normal date. However, foliar application of arginine or Put (1.25 and 2.5 mM) were effective to improve the heat stress by exhibiting significant increments in the growth and all yield parameters in the late sowing plants compared to the untreated control sown at normal date [162]. Polyamine levels can influence synthesis of heat-shock proteins which have important roles in maintaining integrity and properties of cell membranes under HT stress [163]. An ABA-dependent role of polyamines, along with their catabolic product H_2_O_2_, in stomata closure as a protection mechanism under hot and dry climates, by using several techniques, such as DAB, TEM, SEM and DCFH-DA fluorescence. In Spd treated leaf epidermis, Spd induced stomata closure, which was restricted by application of aminoguanidine and guazatine, the two well-known inhibitors of polyamine oxidases. Additionally, post-treatment with Spd, H_2_O_2_ was shown to be localized particularly in guard cells, suggesting the protective role of polyamines and polyamine oxidases in plant temperature/water homeostasis via stomata [164]. Reductions in the activities of POX and IAA oxidase enzymes due to arginine or Put (0.0, 1.25 and 2.5 mM) treated plants prior to HT (35 ± 2 °C) resulted in significant increases in SOD and CAT activities relative to the plants exposed to HT stress alone [149].

Selenium is a trace element, although not recognized as an essential element for plants, proved to serve as stress protectant under various environmental adversities including HT. Djanaguiraman *et al.* [40] investigated the effects of Se foliar spray (75 mg L^−1^) on leaf photosynthesis, membrane stability and antioxidant enzymes activity and grain yield and yield components of grain sorghum (*S. bicolor*) plants grown under HT stress (40/30 °C). They observed that HT stress decreased chl content, chl *a* fluorescence, Pn and antioxidant enzyme activities and increased oxidant production and membrane damage. Decreased antioxidant defense under HT stress resulted in lower grain yield compared with OT (32/22 °C). However, application of Se decreased membrane damage by enhancing antioxidant defense resulting in higher grain yield. The increase in antioxidant enzyme activities and decrease in ROS content by Se was greater in HT than in control. Overall, Se application was significantly increased photosynthetic rate, stomatal conductance and transpiration rate by 13%, 12% and 8%, respectively, compared with the unsprayed control. In addition, foliar spray of Se significantly reduced O_2_^·−^ content, H_2_O_2_ content, MDA level and membrane injury by 11%, 35%, 28% and 18%, respectively, compared with unsprayed plants. Moreover, Se application increased CAT activity in both control and HT stress; however, the maximum increase was observed in HT stress. Across the days of observation, Se application was increased CAT and POX enzyme activity by 26% and 24%, respectively, under HT stress and 9% and 3%, respectively, under control temperature. As a result, Se spray was significantly increased filled seed weight and seed size by 26% and 11%, respectively, over the untreated controls [40]. Many micronutrients application in plants at lower concentration also provided tolerance against HT stress as reviewed by Waraich *et al.* [21].

## 7. Molecular and Biotechnological Strategies for Development of Heat Stress Tolerance in Plants

Along with different physiological and biochemical mechanisms, molecular approaches are boosting to understand the concept of heat stress tolerance in plants very clearly. Plants tolerate such stresses by modulating multiple genes and by coordinating the expression of genes in different pathways [115].

### 7.1. Heat-Shock Proteins (HSPs): Master Players for Heat Stress Tolerance

In general, heat stress is responsible for the up-regulation of several heat inducible genes, commonly referred as “heat shock genes” (HSGs) which encode HSPs and these active products are very much necessary for plant’s survival under fatal HT [165]. High temperature induced constitutive expression of most of these proteins protect intracellular proteins from being denaturation and preserve their stability and function through protein folding; thus it acts as chaperones [166]. The HSPs are extremely heterogeneous in nature and this dynamic protein family is expanding continuously as per the recent researches are going on. The expression of HSPs is restricted to certain developmental stages of plant like seed germination, embryogenesis, microsporogenesis and fruit maturation [167]. In plants, well-characterized HSPs can be grouped into five different families: HSP100 (or ClpB), HSP90, HSP70 (or DnaK), HSP60 (or GroE) and HSP 20 (or small HSP, sHSP) [168]. The HSP70 and HSP60 proteins are among the most highly conserved proteins in nature, consistent with a fundamental role in response to heat stress [169]. There are mutants defective in thermo-tolerance that shows normal induction of HSPs [170]. Plants also have the highest diversity of sHSPs [171] which have very low molecular mass of 12–40 kDa [112]. For better understanding, Table 3 presents the primary molecular functions of major HSPs for heat stress tolerance in plants.

**Figure 6:**
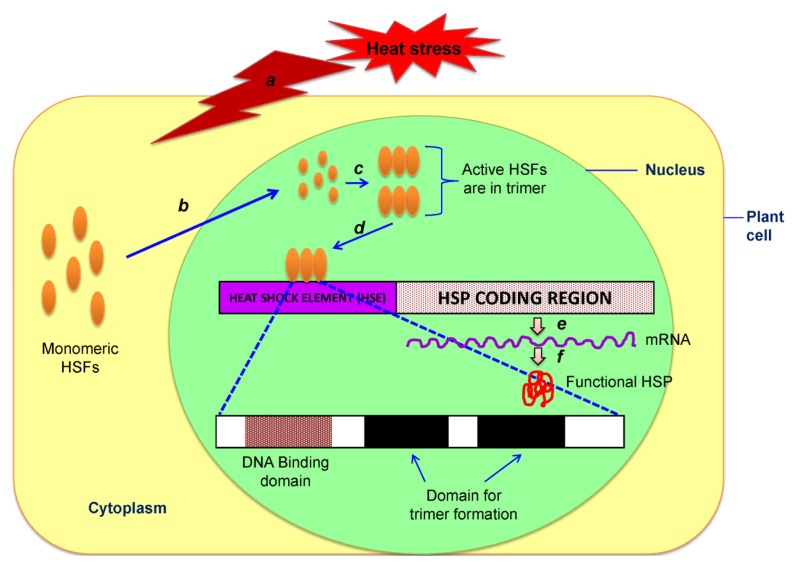
Schematic diagram showing the molecular regulatory mechanism of heat shock proteins based on a hypothetical cellular model. Upon heat stress perceived by the plant cell, (**a**) monomeric heat shock factors (HSFs) are entering into the nucleus; (**b**) from the cytoplasm. In the nucleus, HSF monomers are form active trimer; (**c**) that will bind; (**d**) to the specific genomic region (promoter or heat shock element, HSE) of the respective heat shock gene (HSG). Molecular dissection of the HSF binding region of HSE showing that it is consists of one DNA binding domain and two domains for trimerization of HSFs. Successful transcription (**e**) translation and post-translational modification; (**f**) lead to produce functional HSP to protect the plant cell and responsible for heat stress tolerance.

Due to their thermotolerant nature, the expression of HSP can be induced by heat treatment in the presence of conserved heat shock elements (HSEs) in the promoter region of HSGs, which triggering transcription in response to heat. These *cis*-acting elements (HSE) consist of the palindromic nucleotide sequence (5-AGAANNTTCT-3) that serve as recognizing as well as binding site for heat shock transcription factors or simply heat shock factors (HSFs) [172]. Thermotolerance against heat stress have been accomplished in plants transferred with heat shock regulatory proteins. In most of the plant species, HSFs are constitutively expressed; in normal conditions, these proteins (HSPs) are present as a monomer bound to one of the HSP70 in the cytoplasm. Once the plant has sensed a heat stress (increase in temperature), HSP70 dissociates from cytoplasmic monomeric HSFs and then it enters into the nucleus and form a trimer that can bind with the HSEs [173]. Heat shock factor binding recruits other transcriptional components, resulting in gene expression within minutes in increased temperature (Figure 6). Since all HSGs contain HSE conserved sequence, overexpression of HSF gene intern turned on almost all HSGs and consequently provides protection against heat stress. Although this basic system is universal to eukaryotic cells, it is highly complicated in plants. Unlike animals and yeasts, which may have four or fewer HSFs, plants have been shown to have multiple copies of these genes: tomato has at least 17 and *Arabidopsis* has 21 different HSF genes. These genes have been classified into three groups (classes A, B and C), which are discriminated by features of their flexible linkers and oligomerization domains [172]. Many of the HSFs are heat inducible, suggesting that the specific HSF involved in transcription of a particular gene may vary depending on the timing and intensity of the stress. In general, over expression of plant HSFs can increase plant’s thermo-tolerance, but gene knockouts of individual HSFs tested so far have had little effect on survival at HT. In plants, there are a number of non-HSP transcripts that are upregulated by heat [171]. In particular, the *Arabidopsis* cytosolic ascorbate peroxidase gene (*APX1*) has been shown not only to be heat upregulated, but also to contain a functional heat shock element (HSE) in its 5'-promoter region. In *Arabidopsi*s, *HSFA1a* and *HSFA1b* appear to control the early response of many genes to heat, other HSFs are apparently responsible for the induction of genes expressed later, potentially including heat-inducible HSFs. Interestingly, in tomato, one particular HSF (*HSFA1*) has been proposed to be the ‘master regulator’ of the heat shock response. If this gene is suppressed, normal HSP production does not occur and the plant is extremely sensitive to HTs [174]. Different HSFs have also been shown to act synergistically. Thus, plants appear to have a remarkable ability to finely control the expression of heat induced genes through the HSF system. Some studies also support that there is a positive correlation between the HSP level in the cell and respective stress tolerance [175,176]. Though it is not very much clear that how HSPs confer heat stress tolerance, a recent investigation focused on the *in vivo* function of thermoprotection, governed by sHSPs, is achieved through its assembly into functional stress granules or heat shock granules [177].

Heat shock protein, singly or in form of chaperone, has been implicated in plant cell protection mechanisms under heat stress; they are responsible for protein synthesis, targeting, maturation and degradation, and function in protein and membrane stabilization, and protein renaturation under heat damage condition [178]. Protein denaturation occurs under HT because decreased cellular volume increases the likelihood of degradative molecular interactions. Heat shock proteins maintain and repair companion protein structure and target incorrectly aggregated and non-native proteins for degradation and removal from cells [179]. These proteins primarily function to control the proper folding and conformation of both structural (*i.e*., cell membrane) and functional (*i.e.*, enzyme) proteins, ensuring the correct function of many cellular proteins under conditions of elevated temperature. One such protein, NtHSP70-1, was constitutively overexpressed in tobacco to ascertain its role in plant stress response and tolerance [180]. Liming *et al.* [181] showed that transforming plants with HSP24 from *Trichederma harzianum* was found to confer significantly higher resistance to heat stress when constitutively expressed in *Saccharomyces cerevisiae*. Today’s genetic analyses are aimed to improve high-temperature tolerance level in major crop plants. As heat stress tolerance is a polygenic trait (controlled by different sets of genes), various different components of tolerance are critical at different developmental stages or in different tissues of plant; hence, it shows spacio-temporal mechanism and regulation [182]. Thus, the use of genetic stocks with different degrees of heat tolerance, correlation and co-segregation analyses, molecular biology techniques and molecular markers to identify tolerance QTLs are promising approaches to dissect the genetic basis of plant’s thermotolerance [183].

### 7.2. Genetic Engineering and Transgenic Approaches in Conferring Heat Stress Tolerance in Plants

The adverse effects of heat stress can be mitigated by developing crop plants with improved thermo tolerance using various genetic engineering and transgenic approaches [24]. Constitutive expression of specific proteins has been shown to enhance heat tolerance. In addition to the studies concerning expression of sHSPs/chaperones and manipulation of HSF gene expression, other transgenic plants with varying degrees of heat tolerance have been produced. Surprisingly, however, such experiments have been quite limited compared to the experiments aimed at engineering drought, salt or cold stress tolerance. Lee *et al.* [173] successfully altered the expression level of heat-shock proteins (HSPs) by making a change in the transcription factor (*AtHSF1*) responsible for HSPs in *Arabidopsis* plants and able to produce transgenic HT stress tolerant *Arabidopsis*. It was shown that *AtHSF1* of this plant is constitutively expressed; as in OT condition, its activity for DNA binding, trimer-formation and transcriptional activation of HSP genes are repressed. When *AtHSF1* gene was over expressed, the transcription factor was not active for heat tolerance. However, the fusion of this gene with the *N* or *C* terminus of *gusA* reporter gene (for β-glucuronidase synthesis) produced a fusion protein which was able to trimerize itself and/or with the other HSFs in the absence of heat. Transformation of this fusion protein into *A. thaliana* produced transgenic plants that expressed HSPs constitutive and demonstrated enhanced thermotolerance without requiring prior heat treatment. Malik *et al.* [184] reported the increase in thermotolerant in transgenic carrot cell lines and other plants by constitutive expression of carrot *Hsp17.7* gene driven *CaMV35S* promoter. It has already reported that mitochondrial small HSP (*MT-sHSP*) in tomato has a molecular chaperone function *in vitro* [185] and recently it has been proved that this gene is used for the production of thermotolerant transformed tobacco [186]. Some successful transgenic cases were reported in rice to improve heat tolerance level after the incorporation of *HSP* genes. Katiyar-Agarwal *et al.* [187] were able to successful overexpression of *Arabidopsis Hsp101* gene in transgenic rice to enhance thermotolerance. Moreover, overexpressing rice chloroplast *sHSP* (*Oshsp26*) gene conferred better tolerance to heat stress and other associated oxidative stress in *E. coli* [188]. Overexpression of *sHSP17.7* confers heat tolerance to rice plants [189]. Ono *et al.* [190] transferred *Dnak1* gene (for high salinity tolerance) from the salt-tolerant cyanobacterium *Aphanothece halophytica* to tobacco and successful for its expression to conferred HT tolerance.

Artificial introduction of high levels of the compatible solute GB into *Arabidopsis* through transformation with a bacterial choline oxidase gene engineered to target the protein to the chloroplast was found to significantly enhance germination rates and seedling growth at elevated temperatures [191]. Yang *et al.* [192] suggested the transformation of *BADH* gene in plants for the over production of GB osmolyte that will enhance the heat tolerance. Stability of Rubisco activase (responsible for the activity of rubisco) to HT is essential for its activity maintenance [193]. Thermotolerance is gained by transforming tobacco with Rubisco activase gene for the reversible decarboxylation of Rubisco; such protective mechanism helps to protect the photosynthetic apparatus of plants [194]. Altering fatty acid composition of lipids to increase HT stability of the photosynthetic membrane has also been shown to increase heat tolerance and limits photo-oxidation due to the release of free radicals. Membrane fluidity alterations may also change the perception of the stress through lipid signaling, thus changing the response of protective mechanisms. By silencing the gene encoding chloroplast omega-3 fatty acid desaturase, Murakami *et al.* [195] have been produced transgenic tobacco with altered chloroplast membranes. Such transgenic plants produce comparatively reduced amounts of trienoic fatty acids and more dienoic fatty acids in chloroplasts than the wild type. As measured by O_2_ evolution, photosynthesis appeared to be stabilized against short HT treatments (40 or 45 °C, 5 min) and tobacco plants could grow both under long-term chronic stress as well as short-term acute stress better than wild type. Constitutive expression of an H_2_O_2_ responsive MAPK kinase kinase (MAPKKK) in tobacco (*ANP1*/*NPK1*) was found to protect plants against the lethality in HT (48 °C, 45 min) [196]. Interestingly, an *NPK1*-related transcript was significantly elevated by heat in studies of Rizhsky *et al.* [197]. A report by Shi *et al.* [198] found a modest increase in heat tolerance of *Arabidopsis* plants constitutively expressing the barley *APX1* gene. Doubling the pool of xanthophyll cycle intermediates by over expression of β-carotene hydroxylase protected plants from heat stress under high light conditions [199]. They concluded that the carotenoids were acting by general protection from oxidative stress. Taken together, these data suggest that some form of heat tolerance can be induced through protection of several different systems damaged during heat stress. This is consistent with the interpretation that the acquisition of thermo tolerance involves induction of a number of parallel systems that protect the plant against different types of heat-induced damage. Table 4 summarizes some commonly used transgenes and their functions for making heat stress tolerant transgenic plants. Recently, Grover *et al.* [200] indicated several ways to use transgenic plants in developing HT stress tolerance which may be achieved by overexpressing HSP genes or by altering levels of HSFs that regulate expression of heat shock and non-heat shock genes, overexpression of other trans-acting factors like DREB2A, bZIP28 and WRKY proteins. In addition, genetic improvement of proteins involved in osmotic adjustment, ROS detoxification, photosynthetic reactions, production of PAs and protein biosynthesis process have resulted positive results in developing transgenic plants with HT tolerance.

### 7.3. Omics Approaches in Developing Heat Stress Tolerance

**Figure 7:**
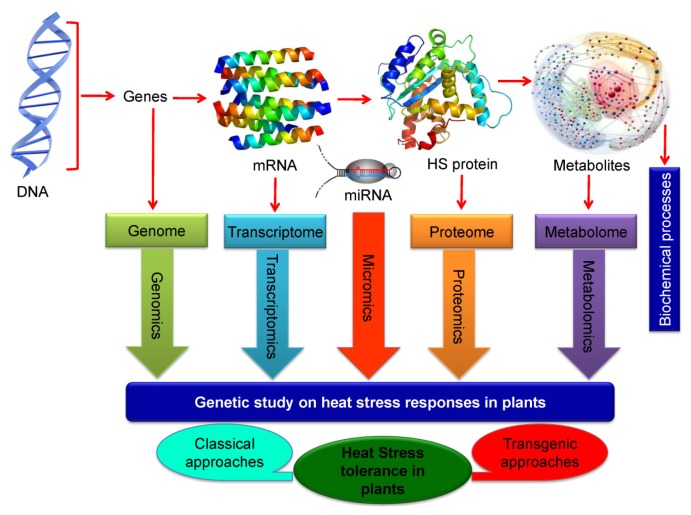
Diagram representing integrated circuit of different “omics” approaches that are connected to each other at molecular genetic level associated with heat stress tolerance in plants.

New advances in “omics” technologies have provided new opportunities and hopes for the identification of transcriptional, translational and post-translational mechanisms and signaling pathways that regulate the plant response(s) to abiotic stress including HT [204,205]. Such omic approaches help to systematic analysis and correlation between the changes in the genome, transcriptome, microme, proteome and metabolome to the variability in plant’s response to temperature extremes and their application to increase the chances of developing stress tolerant plants (Figure 7). DNA is the starting point of all molecular evidences related to heat stress tolerance in plants and contains several heat stress responsive genes in their genome (genomics). A large number of genes with potential roles in heat stress responses have been identified using genetic screens and genome wide expression studies [206]. Transcriptory products (mRNAs), from such genes in the genome, have made their transcriptome (transcriptomics) and then proteome (proteomics) when they translate into the functional proteins (responsible for stress tolerance). In response to developmental and environmental cues, plants employ a post-transcriptional regulation of gene expression by non-protein coding small RNAs or microRNAs (miRNAs) [9]. Micro RNA plays an important functional role in such study and micromics will helps for the better understanding of tolerance. Several heat stress responsive miRNAs have been identified in plants and their role in osmolyte accumulation (osmoprotection) and nutrient starvation response have been established. Stress-upregulated miRNAs may down-regulate their target genes in the onset of thermal stress and act as negative regulators of stress tolerance, while stress down regulated miRNAs may result in accumulation of their target gene mRNAs, which may positively regulate the heat stress tolerance. Overexpression of miRNA-resistant target genes will help to overcome post-transcriptional gene silencing, and thus may lead to better expression of engineered trait in transgenic plants. Understanding the roles of small RNAs in transcriptome homeostasis, cellular tolerance, phenological and developmental plasticity of plants under heat stress and recovery will help genetic engineering of stress tolerance in crop plants [9]. Moreover, proteomes are interlinked in various biochemical processes and will produce several metabolic products (metabolome) under metabolomics platform. Comparing metabolomics between heat and other major categories of abiotic stresses have identified metabolites that are generally important in stress responses or are specific to each stress [207,208]. *Arabidopsis* metabolite profiling reveals that heat stress reduced the toxicity of bioactive compounds like Pro and this event indicating that during the more severe combined stress treatment, sucrose replaces Pro in plants as the major osmoprotectants [209]. When comparing both heat shock and cold shock responses of *Arabidopsis*, comparative metabolomics showed the overlapping nature of major metabolites in respond to heat shock with those metabolites that are under cold shock response [210]. Thus, it is proved that the metabolic network of compatible solutes (Pro, GB, glucose, fructose, galactinol, raffinose, *etc.*) have important roles in tolerance to heat stress. A RNA-binding protein (*ATGRP7*) was identified which increased in response to low temperature stress and opposite when under extreme temperature condition. Its abundance was significantly correlated with glutamine and Pro concentrations. While raffinose and galactinol concentrations were significant markers for temperature responses, their response was independent of the responses of *ATGRP7*, Pro and glutamine [207]. These “omics” approaches are needed for the molecular genetic analyses on HT responses in plants in an integrated fashion. This approach aims to dissect genes in many pathways in order to locate a gene in the stress-response cascade with an outlining on its contribution to tolerance acquisition [115]. Stress tolerance regulation which is determined by chromatin morphology, transcription factor binding and *cis*-regulatory DNA sequences can be inferred by transcript profiling bases mining and the local architecture of respective promoter regions. Heat stress-regulated genes with promoter structures and regulation in cell and tissue level can rely on the increasing number of microarray datasets in present day [182]. After proper combination of transcript analysis and metabolite profile, integrative approaches have been developed and it reflecting that downstream results of altered transcription might be most easily documented in this way [211]. In developing metabolite sensors and databases that will unite transcriptome and metabolome, a prime scientific attention is needed for metabolite analysis [212].

Microarray technology has recently become a powerful tool for the systematic analysis of expression (or transcriptome) profiles of large numbers of genes those are induced or repressed by heat treatment [206,213]. An initial study in *N. tabacum* examined the transcripts of 170 cDNAs from plants drought-stressed with or without simultaneous heat stress [214]. Many unique genes, not upregulated by heat or drought alone, were shown to be up regulated by a combination of these two. They have also used complete genome arrays from *Arabidopsis* to examine transcript changes in response to heat (38 °C, 6 h), drought (70% relative water content) and/or combined treatment. At the single time point tested, they had found 262% increased transcripts in response to heat [197]. Interestingly, among genes, only 29 represented overlap between the two different stresses. However, as in the tobacco study, many transcripts were increased only in response to the combined stresses. Microarray studies are also expanding our knowledge of potential functions required for heat stress tolerance. Recent microarray studies in *Arabidopsis* deficient with *APX* gene, however, have found that certain HSPs are expressed typically under other stress conditions, although expression of HSPs under heat stress occurs normally [215]. This suggests that, while there appear to be distinct pathways for the induction of HSPs during heat stress, there may be some crosstalk between the pathways leading to heat stress tolerance.

## 8. Conclusion and Future Perspective

High temperature stress has become a major concern for crop production worldwide because it greatly affects the growth, development, and productivity of plants. However, the extent to which this occurs in specific climatic zones depends on the probability and period of HT and on the diurnal timing of HT. The present rate of emission of greenhouse gases from different sources is believed responsible for a gradual increase in the world’s ambient temperature, and is resulting in global warming [216]. Therefore, plant responses and adaptation to elevated temperatures, and the mechanisms underlying the development of heat-tolerance, need to be better understood for important agricultural crops. The responses of plants to heat stress have been studied intensively in recent years; however, a complete understanding of thermotolerance mechanisms remains elusive. Temperatures change from season to season and fluctuate daily, which complicates the unambiguous definition of the stress-inducing role of temperature, since the response to various temperatures is determined by a plant’s ability to adapt to different climate regimes. Plant responses to HT also vary across and within species, as well as at different developmental stages.

Under HT conditions, plants accumulate different metabolites (such as antioxidants, osmoprotectants, heat shock proteins [HSPs], *etc.*) and different metabolic pathways and processes are activated. These changes emphasize the importance of physiological and molecular studies to reveal the mechanisms underlying stress responses. In addition, understanding the nature of the signaling cascades as well as the specific genes expressed in response to HT will be valuable for developing stress tolerant plants. Molecular approaches that uncover the response and tolerance mechanisms will pave the way to engineering plants capable of tolerating HT and could be the basis for development of crop varieties capable of producing economic yields under HT [24,106]. At the field level, managing or manipulating cultural practices, such as the timing and methods for sowing, irrigation management, and selection of cultivars and species, can also considerably decrease the adverse effects of HT stress. In recent decades, exogenous applications of protectants such as osmoprotectants, phytohormones, signaling molecules, trace elements, *etc.* have shown beneficial effects on plants growing under HT, due to the growth promoting and antioxidant activities of these compounds [15,16,18,135–137]. Engineering plants to synthesize these compounds may be an alternative way of developing thermotolerance in important crop plants and represents a potentially important area of research on thermotolerance. However, most of the experiments on HT effects currently carried out in different parts of the world are still limited to laboratory conditions and short-term studies only. Field experiments that explore different biochemical and molecular approaches and agronomic management practices are needed to investigate the actual HT responses and their effects on final crop yield.

## Figures and Tables

**Table 1:** Effects of high temperature stress in different crop species.

Crops	Heat treatment	Growth stage	Major effects	References
Chili pepper (*Capsicum annuum*)	38/30 °C (day/night)	Reproductive, maturity and harvesting stage	Reduced fruit width and fruit weight, increased the proportion of abnormal seeds per fruit.	[36]
Rice (*Oryza sativa*)	Above 33 °C, 10 days	Heading stage	Reduced the rates of pollen and spikelet fertility.	[37]
Wheat (*Triticum aestivum*)	37/28 °C (day/night), 20 days	Grain filling and maturity stage	Shortened duration of grain filling and maturity, decreases in kernel weight and yield.	[38]
Wheat (*Triticum aestivum*)	30/25 °C day/night	From 60 DAS to maturity stage	Reduced leaf size, shortened period for days to booting, heading, anthesis, and maturity, drastic reduction of number of grains/spike and smaller grain size and reduced yield.	[39]
Sorghum (*Hordeum vulgare*)	40/30 °C (day/night)	65 DAS to maturity stage	Decreased chlorophyll (chl) content, chl *a* fluorescence, decreased photosystem II (PSII) photochemistry, Pn and antioxidant enzyme activity and increased ROS content, and thylakoid membrane damage, reduced yield.	[40]
Rice (*Oryza sativa*)	32 °C (night temperature)	Reproductive stage	Decreased yield, increased spikelet sterility, decreased grain length, width and weight.	[41]
Maize (*Zea mays*)	35/27 °C (day/night), 14 days	Reproductive stage	Reduced ear expansion, particularly suppression of cob extensibility by impairing hemicellulose and cellulose synthesis through reduction of photosynthate supply.	[42]
Rice (*Oryza sativa*)	25–42.5 °C	Vegetative growth stage	Decrease in the CO_2_ assimilation rate.	[43]
Soybean (*Glycine max*)	38/28 °C (day/night), 14 days	Flowering stage	Decreased the leaf Pn and stomatal conductance (*gs*), increased thicknesses of the palisade and spongy layers, damaged plasma membrane, chloroplast membrane, and thylakoid membranes, distorted mitochondrial membranes, cristae and matrix.	[44]
Tobacco (*Nicotiana tabacum*)	43 °C, 2 h	Early growth stage	Decrease in net photosynthetic rate (Pn), stomatal conductance as well as the apparent quantum yield (AQY) and carboxylation efficiency (CE) of photosynthesis. Reduced the activities of antioxidant enzymes.	[45]
Okra (*Abelmoschus esculentus*)	32 and 34 °C	Throughout the growing period	Reduced yield, damages in pod quality parameters such as fibre content and break down of the Ca-pectate.	[46]
Maize (*Zea mays*)	33–40 °C, 15 days	During Pre-anthesis and silking onwards	Severe effect on plant and ear growth rates.	[47]
Wheat (*Triticum aestivum*)	38 °C, 24 and 48 h	Seedling stage	Decreased chl and relative water content (RWC); diminished antioxidative capacity.	[18]
Wheat (*Triticum aestivum*)	32/24 °C (day/night), 24 h	At the end of spikelet initiation stage	Spikelet sterility, reduced grain yield.	[48]

DAS—Days after sowing.

**Table 2:** Protective effects of exogenous molecules under different heat stress conditions.

Crops	Heat treatments	Protectants	Protective effects	References
*Saccharum officinarum*	42 °C, 48 h	20 mM Pro or GB, 8 h	Restricted the H_2_O_2_ generation, improved K^+^ and Ca^2+^ contents, and increased the concentrations of free Pro	[138]
*Cicer arietinum*	45/40 °C, 10 days	10 μM Pro, 10 days	Reduced membrane injury Improved water and chl content Enhanced activities of antioxidants Reduced oxidative stress Enhance activities of enzymes of carbon metabolism	[139]
*Cicer arietinum*	35/30, 40/35 and 45/40 °C as day/night	10 μM Pro, GB and Tre	Increased growth Less oxidative damages Decreased MDA and H_2_O_2_ contents	[140]
*Oryza sativa*	35 °C, 48 h	0.5 mM SA, 24 h	Decreased electrolyte osmosis Reduced MDA content and O_2_^·−^ production rate	[141]
*Vitis vinifera*	43 °C, 24 h	100 μM SA, 24 h	Higher Rubisco activity Increased PSII function Increased photosynthesis	[142]
*Brassica juncea*	47 ± 5 °C	0.5 & 1 μM ABA, 4 h	Decreased seedling mortality Increased growth	[143]
*Cicer arietinum*	35/30, 40/35 and 45/40 °C as day/night	2.5 μM ABA	Increased growth Less oxidative damages Decreased MDA and H_2_O_2_ contents	[140]
*Phaseolus vulgaris*	34.7 to 35.2 °C	25, 50 mg L^−1^ BRs spray	Increased vegetative growth, total yield and quality of pods Increased the total phenolic acids in the pod	[144]
*Brassica juncea*	40 °C, 5 h × 3 days	1 μM 24-EBL, 8 h	Better growth Increased protein content Enhanced antioxidant defense	[145]
*Brassica juncea*	47 ± 5 °C	100 μM IAA, 4 h	Decreased seedling mortality Increased growth	[143]
*Brassica juncea*	47 ± 5 °C	100 μM GA, 4 h	Decreased seedling mortality Increased growth	[143]
*Vitis vinifera*	42 °C, 12 & 18 h	50 μM JA, 6 h	Upregulation of the activities of antioxidant enzymes	[146]
*Brassica juncea*	47 ± 5 °C	50 and 100 μM kinetin	Decreased seedling mortality Increased growth	[143]
*Phragmites communis*	45 °C, 2 h	100 μM SNP and SNAP, 24 h	Decreased H_2_O_2_ and MDA contents. Increased activities of SOD, CAT, APX and POD	[147]
*Phaseolus radiatus*	45 °C, 90 min	150 μM SNP, 60 min	Increased the activities of CAT, SOD and POD	[148]
*Triticum aestivum*	35 ± 2 °C, 4 or 8 h	Arginine or Put (0.0, 1.25 and 2.5 mM), 4 or 8 h	Increased SOD and CAT activities, increased DNA and RNA contents, reduced MDA level	[149]
*Solanum lycopersicum*	33/27 °C, 16/8 h (light/dark)	Spd, 1 mM as pretreatment	Increase in the expression of Eth-related genes, PA biosynthesis genes, hormone pathways genes, and oxidation reduction genes	[150]
*Gossypium hirsutum*	38 °C up to flowering stage	10 mM Put, 24 h prior to anthesis	Increased endogenous Put content and seeds/cotton boll	[151]
*Triticum aestivum*	45 °C in germinated seeds, 2 h	Put, 10 μM	Elevated activities of enzymatic and non-enzymatic antioxidants and DAO and PAO, reduced lipid peroxides in root and shoot	[152]
*Sorghum bicolor*	40/30 °C, 45 days	75 mg L^−1^ Na_2_SeO_4_ foliar spray	Decreased membrane damage Enhanced antioxidant defense Increased grain yield	[40]

**Table 3:** An outline of basic function of major classes of heat shock proteins in plant system for heat stress tolerance.

Major classes of heat shock protein	Functions
HSP100	ATP-dependent dissociation and degradation of aggregate protein
HSP90	Co-regulator of heat stress linked signal transduction complexes and manages protein folding. It requires ATP for its function
HSP70, HSP40	Primary stabilization of newly formed proteins, ATP-dependent binding and release
HSP60, HSP10	ATP-dependent specialized folding machinery
HSP20 or small HSP (sHSP)	Formation of high molecular weight oligomeric complexes which serve as cellular matrix for stabilization of unfolded proteins. HSP100, HSP70 and HSP40 are needed for its release

**Table 4:** List of transgenic plants, heat stress linked transgenes and their responsible role for enhancing plants towards stress tolerance.

Transgenic plants	Transgenes	Function of transgenes	References
*Z. mays* and *O. sativa*	*Hsp100*, *Hsp101* from *A. thaliana*	HSP synthesis for HT tolerance	[114,187]
*A. thaliana*	*Hsp70*	HSP synthesis for thermotolerance	[201]
*N. tabacum*	*Fad 7* from *N. tabacum* and *O. sativa*	Desaturation of fatty acids (trienoic fatty acids and hexa-decatrienoic acid) that increased the level of unsaturated fatty acids and provide HT tolerance	[195,202]
*Daucus carota*	*Hsp17.7* from *D. carota*	Synthesis of sHsp	[184,189]
*N. tabacum*	*TLHS1*	Synthesis of sHSP (Class I)	[203]
*A. thaliana*	*AtHSF1*	Heat shock transcription factor HSF1::GUS (β-glucuronidase) fusion and such modification will increase HSP production in large scale with small investment of HSFs	[173]
*A. thaliana*	*gusA*	β-glucuronidase synthesis and bind with HSFs to form active trimer	[173]
*N. tabacum*	*MT-sHSP* from *L. esculentum*	Molecular chaperone function *in vitro*	[185,186]
*N. tabacum*	*Dnak1* from *Aphanothece halophytica*	High temperature tolerance	[190]
*N. tabacum*	*BADH* (betain aldehyde dehydrogenase) from *Spinacia oleracea*	Over production of GB osmolyte that will enhance the heat tolerance	[192]
*A. thaliana*	*Cod A* (choline oxidase A) from *A. globiformis*	Glycine betaine systhesis for tolerance to HT during imbibition and seedling germination	[191]
*N. tabacum*	*ANP1*/*NPK1*	H_2_O_2_ responsive MAPK kinase kinase (MAPKKK) production to protect against the lethality in HT	[196]
*A. thaliana*	Ascorbate peroxidase (*APX1* from *P. sativum* and *HvAPX1* from *H. vulgare*)	H_2_O_2_ detoxification and conferred heat tolerance	[198]
